# Prognosis of patients with esophageal squamous cell carcinoma after esophagectomy using the log odds of positive lymph nodes

**DOI:** 10.18632/oncotarget.5366

**Published:** 2015-09-29

**Authors:** San-Gang Wu, Jia-Yuan Sun, Li-Chao Yang, Juan Zhou, Feng-Yan Li, Qun Li, Huan-Xin Lin, Qin Lin, Zhen-Yu He

**Affiliations:** ^1^ Xiamen Cancer Center, Department of Radiation Oncology, the First Affiliated Hospital of Xiamen University, Xiamen, People's Republic of China; ^2^ Sun Yat-sen University Cancer Center, State Key Laboratory of Oncology in South China, Department of Radiation Oncology, Collaborative Innovation Center of Cancer Medicine, Guangzhou, People's Republic of China; ^3^ Department of Basic Medical Science, Medical College, Xiamen University, Xiamen, People's Republic of China; ^4^ Xiamen Cancer Center, Department of Obstetrics and Gynecology, the First Affiliated Hospital of Xiamen University, Xiamen, People's Republic of China

**Keywords:** esophageal squamous cell carcinoma, lymph nodes, log odds of positive lymph nodes, lymph node ratio, prognosis

## Abstract

To compare the log odds of positive lymph nodes (LODDS) with the number of positive lymph nodes (pN), lymph node ratio (LNR), removed lymph node (RLN) count, and negative lymph node (NLN) count in determining the prognosis of patients with esophageal squamous cell carcinoma (ESCC) after esophagectomy. The records of patients with ESCC who received esophagectomy were retrospectively reviewed. The log-rank test was used to compare curves for overall survival (OS), and Cox regression analysis was performed to identify prognostic factors. The prognostic performance of the different lymph node staging systems were compared using the linear trend chi-square test, likelihood ratio chi-square test, and Akaike information criterion. A total of 589 patients were enrolled. Univariate Cox analysis showed that pN stage, LNR, RLN count, NLN count, and the LODDS were significantly associated with OS (*p* < 0.05 for all). Multivariate Cox analysis adjusted for significant factors indicated that LODDS was independent risk factor on overall survival (OS), and a higher LODDS was associated with worse OS (hazard ratio = 3.297, 95% confidence interval: 2.684–4.050, *p* < 0.001). The modified Tumor-LODDS-Metastasis staging system had better discriminatory ability, monotonicity, and homogeneity, and better optimistic prognostic stratification than the Tumor-Node-Metastasis staging system in determining the prognosis of patients with ESCC. The LODDS staging system was superior to other lymph node classifications in determining the prognosis of patients with ESCC after esophagectomy. LODDS may be incorporated into esophageal staging system if these results are eventually confirmed by other studies.

## INTRODUCTION

Esophageal cancer (EC) is a common malignancy of the gastrointestinal system, and esophageal squamous cell carcinoma (ESCC) is the major type of EC in China [1, 2]. Currently, the pathologic nodal (pN) stage of EC is determined by the number of positive lymph nodes (PLNs) [3]. However, inaccurate lymph node dissection and pathological evaluation may cause understaging of pN stage and inappropriate treatment, the so-called phenomenon of stage migration [4, 5]. There are several nodal staging systems which have been proposed and investigated to provide a more accurate nodal staging system, including the lymph node ratio (LNR), the number of removed lymph nodes (RLNs), and the number of negative lymph nodes (NLNs) [6–13]. The number of RLNs is an important indicator of the accuracy of staging. However, the therapeutic and prognostic value of the number of RLNs remains controversial [7, 14–16]. In addition, for node-negative EC patients, the LNR status is the same as pN0 classification, and thus LNR does not provide a more accurate prognosis than Tumor-Node-Metastasis (TNM) staging. Moreover, several studies have suggested that a higher number of NLNs is associated with better survival [9–11].

Log odds of positive lymph nodes (LODDS), defined as the log of the ratio between the probability of the number of PLNs and the number of NLNs [16–20]. The LODDS has been determined as novel prognostic factor in patients with gastric cancer, colorectal cancer, and pancreatic cancer [17–23]. Until now, no study accessing the prognostic value of the LODDS in patients with ESCC has been reported. Thus, the aim of this study was to compare the prognostic value of the LODDS with the pN stage, LNR, NLN count, and RLN count in patients with ESCC after esophagectomy.

## RESULTS

### Patient characteristics

A total of 589 patients with a median age of 57 years (range: 30–82 years) were identified. The clinical characteristics of these patients are shown in Table 1. Most patients were male (75.2%, *n* = 443). Most patients were in stage pT3 (66.9%, *n* = 394), 29.2% patients (*n* = 172) in stage pT2 (*n* = 172), and 3.9% patients (*n* = 23) in stage pT1.

**Table 1:** Characteristics of 589 patients with esophageal squamous cell carcinoma

Characteristic	*n* (%)
Age (years)	
≤60	471 (80.0)
>60	118 (20.0)
Sex	
Male	443 (75.2)
Female	146 (24.8)
Tumor location	
Upper third	41 (7.0)
Middle third	248 (42.1)
Lower third	300 (50.9)
Tumor stage	
pT1	23 (3.9)
pT2	172 (29.2)
pT3	394 (66.9)
Node stage	
pN0	327 (55.5)
pN1	153 (26.0)
pN2	88 (14.9)
pN3	21 (3.6)
Histologic grade^a^	
G1	108 (18.3)
G2	302 (51.3)
G3	179 (30.4)
Number of RLNs (n)	
≤14	303 (51.4)
>14	286 (48.6)
Number of NLNs (n)	
≤13	316 (53.7)
>13	273 (46.3)
LNR	
≤0.14	236 (40.1)
>0.14	353 (59.5)
LODDS	
LODDS 1	154 (26.1)
LODDS 2	151 (25.6)
LODDS 3	142 (24.1)
LODDS 4	142 (24.1)
Number of field dissected	
Two-field	453 (77.0)
Three-field	136 (23.0)
Adjuvant chemotherapy	
No	532 (90.3)
Yes	57 (9.7)
Adjuvant radiotherapy	
No	560 (95.1)
Yes	29 (4.9)

a G1 = well differentiated; G2 = moderately differentiated; G3 = poorly differentiated. RLNs, removed lymph nodes; NLNs, negative lymph nodes; LNR, lymph node ratio; LODDS, log odds of positive lymph nodes.

Two-field lymphadenectomy was performed in 453 patients (76.9%), and three-field lymphadenectomy in 136 patients (23.1%). The median number of RLNs was 14 (range: 2–55). There were 48.6% patients (*n* = 286) had more than 14 RLNs, and 303 patients (51.4%) had 14 or fewer RLNs. Overall, 55.5% of patients (*n* = 327) had node-negative disease, and 44.5% (*n* = 262) had nodal metastases. Among patients with nodal metastases, the median number of PLNs was 2 (range: 1–20). There were 153 patients (26.0%) in stage pN1, 88 patients (14.9%) in stage pN2, and 21 patients (3.6%) in stage pN3. The median LNR was 0.14 (range: 0.02–1.00).

The median LODDS value was −1.17 (range: −2.05 − 0.66). Given that the LODDS value was a continuous variable, we examined the LODDS value as a categorical variable based on quartiles. Thus, there were 154 patients in LODDS 1 (range: −2.05 to −1.46), 151 patients in LODDS 2 (range: −1.45 to −1.17), 142 patients in LODDS 3 (range: −1.16 to −0.73), and 142 patients in LODDS 4 (range: −0.72 to 0.66) (Table 1).

### Impact of lymph node status on OS

VIF values for pN stage, LNR, RLN count, NLN count, and LODDS were 5.428, 2.998, 3.988, 4.200, and 5.047, respectively. None of them indicated a significant problem with multicollinearity.

Univariate Cox survival analysis showed that pN stage, LNR, RLN count, NLN count, and LODDS were significant prognostic factors for OS (*p* < 0.001 for all). Other significant prognostic factors included sex, pT stage, histologic grade, adjuvant radiotherapy, and adjuvant chemotherapy (*p* < 0.05 for all) (Table 2).

**Table 2:** Univariate analysis of prognostic factors influencing the survival of ESCC patients

Characteristic	HR	95%CI	*p*
Age (continuous variable)	1.002	0.990–1.013	0.777
Sex	0.745	0.579–0.958	0.022
Tumor location	0.986	0.833–1.165	0.865
Tumor stage	1.596	1.298–1.962	<0.001
Histological grade	1.369	1.172–1.599	<0.001
Node stage	1.915	1.708–2.147	<0.001
Number of field dissected	0.967	0.756–1.238	0.792
RLN count (continuous variable)	0.981	0.969–0.994	0.004
LNR (continuous variable)	23.126	13.633–39.230	<0.001
NLN count (continuous variable)	0.964	0.951–0.978	<0.001
LODDS (continuous variable)	3.460	2.839–4.216	<0.001
Adjuvant chemotherapy (yes vs. no)	1.639	1.182–2.271	0.003
Adjuvant radiotherapy (yes vs. no)	1.542	1.002–2.375	0.049

HR, hazard ratio; CI, confidence interval; RLN, removed lymph node; LNR, lymph node ratio; NLN, negative lymph node; LODDS, log odds of positive lymph nodes.

Multivariate Cox analysis adjusted for significant factors from the univariate analysis was used to assess the association of survival with pN stage (Model 1), LNR (Model 2), RLN count (Model 3), NLN count (Model 4), LODDS (Model 5), separately, and combined with the five lymph node classifications (Model 6). In Models 1–4, pN stage, LNR, RLN count, and NLN count was significantly associated with OS. In Model 5, LODDS was associated with OS (hazard ratio [HR] = 3.326, 95% confidence interval [CI]: 2.175–4.075, *p* < 0.001), but pN stage exhibited no effect on OS (HR 1.182, 95% CI 0.960–1.457, *p* = 0.116). In Model 6, we found the LODDS was independent risk factor on OS, a higher LODDS was associated with worse OS (HR = 3.326, 95%CI: 2.715–4.075, *p* < 0.001), but pN stage, LNR, RLN count, and NLN count exhibited no effect on OS (*p* > 0.05) (Table 3). Other significant prognostic factors included sex, pT stage and histological grade (Table 3).

**Table 3:** Multivariate analysis of prognostic factors influencing the survival of ESCC patients

Characteristic	HR	95%CI	*p*
**Model 1**			
Sex	0.714	0.554–0.919	0.009
Tumor stage	1.348	1.094–1.661	0.005
Histological grade	1.210	1.036–1.414	0.016
Adjuvant chemotherapy	0.907	0.642–1.281	0.579
Adjuvant radiotherapy	1.172	0.753–1.824	0.481
Node stage	1.819	1.612–2.053	<0.001
**Model 2**			
Sex	0.695	0.539–0.896	0.005
Tumor stage	1.327	1.075–1.637	0.008
Histological grade	1.177	1.005–1.378	0.043
Adjuvant chemotherapy	0.886	0.625–1.255	0.494
Adjuvant radiotherapy	1.159	0.744–1.807	0.514
Node stage	1.503	1.249–1.810	<0.001
LNR (continuous variable)	3.938	1.506–10.297	0.005
**Model 3**			
Sex	0.685	0.532–0.883	0.003
Tumor stage	1.333	1.082–1.644	0.007
Histological grade	1.178	1.009–1.376	0.038
Adjuvant chemotherapy	1.049	0.739–1.488	0.790
Adjuvant radiotherapy	1.099	0.704–1.715	0.679
Node stage	1.947	1.718–2.206	<0.001
RLN count (continuous variable)	0.968	0.954–0.981	<0.001
**Model 4**			
Sex	0.679	0.527–0.875	0.003
Tumor stage	1.329	1.078–1.639	0.008
Histological grade	1.174	1.005–1.371	0.044
Adjuvant chemotherapy	1.047	0.737–1.486	0.799
Adjuvant radiotherapy	1.082	0.692–1.691	0.730
Node stage	1.807	1.601–2.038	<0.001
NLN count (continuous variable)	0.967	0.953–0.980	<0.001
**Model 5**			
Sex	0.720	0.559–0.926	0.011
Tumor stage	1.314	1.065–1.620	0.011
Histological grade	1.138	0.972–1.332	0.109
Adjuvant chemotherapy	0.924	0.653–1.307	0.655
Adjuvant radiotherapy	1.119	0.718–1.743	0.620
Node stage	1.182	0.960–1.457	0.116
LODDS (continuous variable)	3.326	2.715–4.075	<0.001
**Model 6**			
Sex	0.720	0.559–0.926	0.011
Tumor stage	1.314	1.065–1.620	0.011
Histological grade	1.147	0.979–1.344	0.090
Adjuvant chemotherapy	0.994	0.691–1.429	0.974
Adjuvant radiotherapy	1.068	0.679–1.680	0.775
Node stage	1.287	0.862–1.920	0.217
LNR (continuous variable)	0.363	0.073–1.811	0.217
RLN count (continuous variable)	1.037	0.944–1.139	0.444
NLN count (continuous variable)	0.952	0.863–1.050	0.327
LODDS (continuous variable)	3.326	2.715–4.075	<0.001

HR, hazard ratio; CI, confidence interval; RLN, removed lymph node; LNR, lymph node ratio; NLN, negative lymph node; LODDS, log odds of positive lymph nodes.

### Prognostic impact of the LODDS on OS

**Figure 1:**
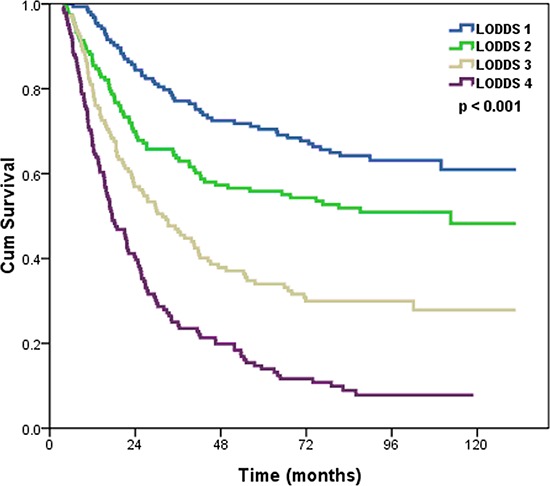
Effects of the LODDS on the survival of ESCC patients

The median follow-up time was 39.2 months (range: 4–131 months) in all patients, and 92.6 months (range: 6–131 months) in surviving patients. A total of 353 patients died, 338 of whom died of ESCC-related diseases, and 15 died of other diseases. The 5-year and 10-year OS was 44.3% and 37.0%, respectively, and the median survival time was 42.2 months. The 5-year OS rates were 70.4%, 55.8%, 34.0%, and 14.0% in LODDS 1, LODDS 2, LODDS 3, and LODDS 4, respectively (*p* < 0.001) (Figure 1).

**Figure 2:**
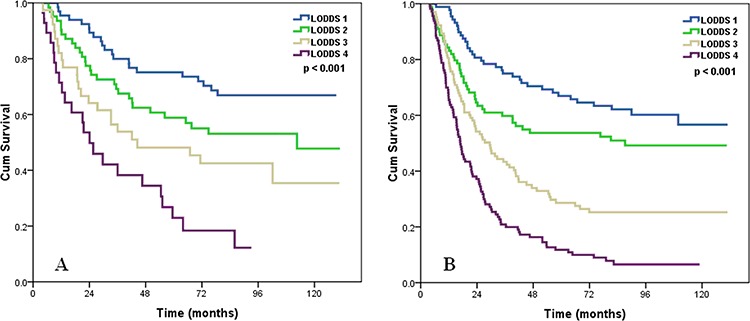
Effects of the LODDS on the survival of ESCC patients with pT1–2 stage A, pT3 stage B

We determined whether the influence of LODDS staging system on OS was modified by the pT stage. In both pT1–2 and pT3 patients, LODDS staging system was significantly associated with OS (log rank *p* < 0.001 for pT1–2, and *p* < 0.001 for pT3 patients) (Figure 2A–2B).

**Figure 3:**
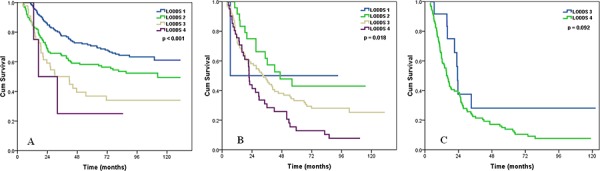
Effects of the LODDS on the survival of ESCC patients with pN0 stage A, pN1 stage B, pN2-3 stage C

We examined the prognostic effect of the LODDS staging system according to different pN stage. In both pN0 (log rank *p* < 0.001) and pN1 patients (log rank *p* = 0.018), LODDS staging system was significantly associated with OS (Figure 3A–3B). There were trends for prognostic effect of the LODDS staging system in pN2–3 patients, but these were not statistically significant (log rank *p* = 0.092) (Table 3C).

**Figure 4:**
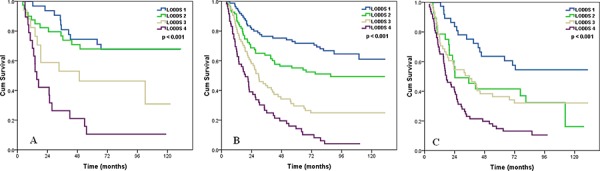
Effects of the LODDS on the survival of ESCC patients with G1 A, G2 B, and G3 C. disease

We then examined the influence of LODDS classifications on OS according to histologic grade. The effect of LODDS classifications significantly difference across any histologic grade group (log rank *p* < 0.001 for G1 patients, *p* < 0.001 for G2 patients, and *p* = 0.027 for G3 patients) (Figure 4A–4C).

**Figure 5:**
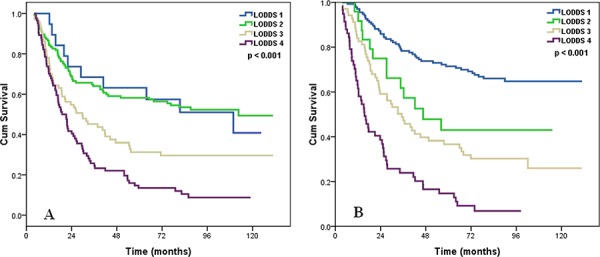
Effects of the LODDS on the survival of ESCC patients with removed lymph node count ≤ 14 A, and removed lymph node count > 14 B

We finally compared the OS rates of patients in different LODDS classifications according to the number of RLNs. The prognostic effect of LODDS classifications was also found in patients with ≤ 14 RLNs (log rank *p* < 0.001) and more than 14 RLNs (log rank *p* < 0.001) (Figure 5A–5B).

### Tumor-LODDS-Metastasis (TLM) staging

We used the likelihood ratio chi-square test, the linear trend chi-square test, and the AIC analysis to compare the prognostic performance of the different lymph node staging systems. Of note, the LODDS staging system performed superiorly to the other lymph node staging systems (Table 4).

**Table 4:** Prognostic performance of different lymph node staging systems

Lymph node staging system	Linear trend chi-square	Likelihood ratio chi-square	AIC
pN stage (categorical)	76.144	91.078	1884.248
LNR (categorical)	58.814	68.074	1944.127
RLN count (categorical)	4.349	4.359	1979.902
NLN count (categorical)	17.197	17.267	1983.912
LODDS (categorical)	99.569	110.654	1872.078

AIC, Akaike information criterion; LNR, lymph node ratio; RLN, removed lymph node; NLN, negative lymph node; LODDS, log odds of positive lymph nodes.

**Figure 6:**
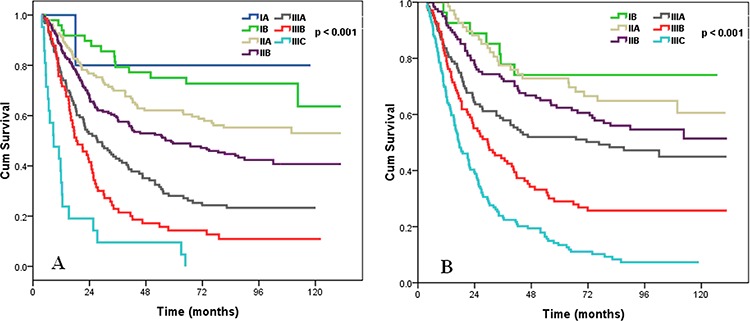
Survival curves of ESCC patients according to AJCC/UICC TNM staging system A, and TLM staging system B

As expected, analysis of patients by cancer stage from the 2009 UICC/AJCC classification system indicated poorer OS in patients with more advanced cancer (*p* < 0.001) (Figure 6A). We developed a Tumor-LODDS-Metastasis (TLM) staging system by replacing the pN stage with the 4 different LODDS classifications. In this new staging system, there was no patient with stage IA. Survival analysis showed that the TLM staging system was also effective in predicting the survival of patients with ESCC (*p* < 0.001) (Figure 6B).

We used the likelihood ratio chi-square test, the linear trend chi-square test, and the AIC analysis to compare the prognostic performance of the TNM and TLM staging systems. The results showed that the TLM staging system had a better prognostic value than the 2009 UICC/AJCC staging system for all 3 tests (Table 5). These results indicated that the new TLM staging system considering the LODDS classification rather than pN stage, is effective in determining the prognosis of patients with ESCC.

**Table 5:** Comparison of the prognostic performances of the TLM staging system and the TNM staging systems

Staging system	Linear trend chi-square	Likelihood ratio chi-square	AIC
TLN	100.21	115.487	1938.928
TNM	85.12	96.162	1940.204

AIC, Akaike information criterion; TLM, Tumor-LODDS-Metastasis; TNM, Tumor-Node- Metastasis.

## DISCUSSION

We examined 589 patients who received radical surgery of ESCC, and compared the prognostic performance of different lymph node staging systems in determination of the OS. The results showed that LODDS was independently associated with OS and that it had better prognostic value than other lymph node staging systems, including pN stage, LNR, NLN count, and RLN count.

Lymph node status is one of the most important factors used to predict survival in ESCC patients. In the original TNM staging system, lymph nodes were simply categorized as absent (N0) or present (N1) [29]. However, there is increasing interest in using the detailed status of lymph nodes for determining the prognosis of patients with EC. The staging system for EC from the 7^th^ edition of the AJCC/UICC reclassified the pN stage according to the number of PLNs [3]. However, the number of RLNs affects the number of PLNs, so staging by PLN count alone has limited use because it may lead to stage migration [4, 5]. In recent years, several nodal staging systems including LNR, RLN count, and NLN count indicated that these parameters had prognostic value for EC patients [6–13]. The LODDS value is a novel indicator of lymph node status that has the potential to further improve the accuracy of pN classification and thereby provide a more reliable and accurate prognosis.

Recent studies that considered the LODDS focused on colorectal cancer, gastric cancer, and pancreatic cancer. These studies showed that use of the LODDS may help to provide a more accurate prognosis of such patients, and that it was superior to other systems used for the staging of lymph nodes [17–23]. To the best of our knowledge, the present study is the first to confirm the prognostic performance of the LODDS in ESCC.

The univariate analysis of the present study indicated that all five lymph node classifications had significant prognostic value. We used multivariate analysis to compare the performances of these different classifications. In particular, we used 6 multivariate models, including all of the five lymph node classifications (pN, LNR, RLNS, NLNs, and LODDS). In the first 4 classifications, pN, LNR, RLNs, and NLNs were significantly and independently associated with OS. In model 5, the LODDS classification was superior to pN classification. In Model 6, we used all five lymph node classifications together in the multivariate analysis, and the results showed that the LODDS classification was superior to these other lymph node staging systems. With that, we believe that the LODDS classification is more likely to minimize the effects of stage migration. In particular, the prognostic value of LODDS of ESCC patients was not significantly different by the extent of lymph node dissection.

In this study, by using the TNM staging system for esophageal cancer as a foundation, we replaced the N stage with the LODDS classification to establish a novel TLM staging system. The results indicated that this new staging system had a better performance than the TNM staging system for the likelihood ratio chi-square test, the linear trend chi-square test, and the AIC. Thus, we suggest that investigators consider incorporation of the LODDS into the lymph node staging used for ESCC in future.

The current study had several limitations that should be considered. First, this was a single-center retrospective analysis. Thus, prospective studies are needed to confirm our results. Second, we excluded patients with stage IV EC, so the relevance of our findings to these patients is unclear. In addition, we used cut-off points of LODDS classification determined according to the interquartile range. To date, no study has investigated the use of LODDS classification in EC, and so the optimal cut-off points have not yet been established.

In conclusion, our findings indicate that the LODDS is independently and significantly associated with survival of patients with ESCC, and that its prognostic value is superior to the other lymph node staging systems including pN stage, LNR, RLN count, and NLN count. LODDS may be incorporated into esophageal staging system if these results are eventually confirmed by other studies.

## MATERIALS AND METHODS

### Patients

We retrospectively reviewed the records of patients with ESCC who received radical esophagectomy from October 2002 to March 2007 in Sun Yat-Sen University Cancer Center. The inclusion criteria were: *(i)* resectable thoracic ESCC with radical esophagectomy and lymph node dissection; *(ii)* staging as pT1–3N0–3M0 according to the 7th edition of the American Joint Committee on Cancer and the Union for International Cancer Control (UICC/AJCC) TNM staging system; *(iii)* negative surgical margin (R0); *(iv)* no preoperative radiotherapy or chemotherapy. Patients died within 3 months after esophagectomy were excluded. This study was approved by the ethics committee of Sun Yat-Sen University Cancer Center. All patients provided written consent for storage of their information in the hospital database and for use of this information in our research.

### Patient characteristics and lymph node status

Patient clinicopathologic factors including sex, age, pathologic tumor (pT) stage, pN stage, histologic grade, tumor location, LNR, RLN count, NLN count, and LODDS were used to assess the risks of overall mortality. The pT and pN stages were determined according to the 7^th^ edition of the AJCC/UICC staging system. The total number of RLNs was the number of dissected lymph nodes from the cervical, intrathoracic, and abdominal regions. The number of NLNs was obtained by subtracting the number of PLNs from the total number of RLNs. The LNR was defined as the ratio of the number of PLNs to the number of RLNs. The LODDS was defined as log_10_([PLNs + 0.5]/[NLNs + 0.5]), in which 0.5 was added to both the numerator and the denominator to avoid an infinite number [24].

### Surgical procedures

Surgery was performed according to our previously reported [6, 11]. Briefly, all patients received radical esophagectomy and lymphadenectomy. The most common surgical approaches included the left thoracotomy, the Ivor-Lewis approach, and the cervico-thoraco-abdominal procedure. Extensive lymph node dissection in the posterior mediastinum and abdomen was routinely performed. The lymph nodes in the middle and lower mediastinum, including the periesophageal, parahiatal, subcarinal, and aortopulmonary nodes were systematically dissected. In the abdomen, an upper abdominal and retroperitoneal lymph nodes dissection was performed including the celiac, splenic, common hepatic, left gastric, lesser curvature, and parahiatal nodes. Cervical lymphadenectomy was not routinely undertaken. Three-field lymphadenectomy was performed in those with suspected supraclavicular lymph node metastasis.

### Examination of lymph nodes

All resected lymph nodes were submitted for pathologic assessment. The pathologists evaluated the resected lymph nodes, which were separately labeled by the surgeons in a routine manner. Lymph node metastasis was evaluated by hematoxylin and eosin (HE) staining. The number of RLNs was the sum of the cervical, intrathoracic, and abdominal lymph nodes.

### Follow-up and survival endpoints

Follow-up after surgery consisted of a hospital visit, telephone call, or mail. In general, patients were scheduled for clinical follow-up every 3 to 6 months, including a physical examination, laboratory tests of complete blood counts, liver function test, chest radiography, ultrasonography, esophagography, and endoscopy. In addition, a fluorine-18-fluorodeoxyglucose positron emission tomography scan was performed if necessary. The endpoint of this study was overall survival (OS). OS was calculated as the time from the date of diagnosis to the date of death from any cause or the date of the last follow-up.

### Statistical analysis

All data were analyzed using the SPSS statistical software package (version 16.0; IBM Corporation, Armonk, NY, USA). The χ^2^ and Fisher's exact probability tests were used to assess differences in qualitative data. Recognizing that RLN count, NLN count, and LODDS may have been incompletely counted or affected by natural inter-individual variation in nodal distribution, there were examined as a categorical variable based on quartiles. The optimal cut-off point of RLN count and NLN count was 14 and 13, respectively, according to our previously reported [9]. The survival curves were plotted by the Kaplan-Meier method and *p*-values were calculated using the log rank test. Cox regression analysis was performed to identify significant prognostic factors. A *p*-value less than 0.05 was considered statistically significant.

The variance inflation factor (VIF) was used to verify the multicollinearity of pN stage, LNR, RLN count, NLN count, and LODDS, a VIF of 10 and above indicates a multicollinearity problem. The prognostic performances of the different lymph node staging systems were compared in terms of homogeneity, discriminatory ability, and monotonicity. We used the likelihood ratio chi-square test, the linear trend chi-square test, and the Akaike information criterion (AIC) within the Cox regression model to compare performances among different lymph node staging systems; the likelihood ratio chi-square test to measure homogeneity, the linear trend chi-square test to measure the discriminatory powers and monotonicities across categories, and the AIC to measure the optimistic prognostic stratifications [25–28]. A higher likelihood ratio chi-square score means better homogeneity, higher linear trend chi-square scores show better discriminatory ability and monotonicity, and smaller AIC values represent better optimistic prognostic stratification.

## References

[1] Chen W, Zheng R, Zhang S, Zhao P, Li G, Wu L, He J (2013). The incidences and mortalities of major cancers in China, 2009. Chin J Cancer.

[2] Siegel R, Ma J, Zou Z, Jemal A (2014). Cancer statistics. CA Cancer J Clin.

[3] Rice TW, Rusch VW, Ishwaran H, Blackstone EH (2010). Cancer of the esophagus and esophagogastric junction: data-driven staging for the seventh edition of the American Joint Committee on Cancer/International Union Against Cancer Cancer Staging Manuals. Cancer.

[4] Twine CP, Lewis WG, Morgan MA, Chan D, Clark GW, Havard T, Crosby TD, Roberts SA, Williams GT (2009). The assessment of prognosis of surgically resected oesophageal cancer is dependent on the number of lymph nodes examined pathologically. Histopathology.

[5] Kong SH, Lee HJ, Ahn HS, Kim JW, Kim WH, Lee KU, Yang HK (2012). Stage migration effect on survival in gastric cancer surgery with extended lymphadenectomy: the reappraisal of positive lymph node ratio as a proper N-staging. Ann Surg.

[6] He Z, Wu S, Li Q, Lin Q, Xu J (2013). Use of the metastatic lymph node ratio to evaluate the prognosis of esophageal cancer patients with node metastasis following radical esophagectomy. PLoS One.

[7] Altorki NK, Zhou XK, Stiles B, Port JL, Paul S, Lee PC, Mazumdar M (2008). Total number of resected lymph nodes predicts survival in esophageal cancer. Ann Surg.

[8] Yang HX, Xu Y, Fu JH, Wang JY, Lin P, Rong TH (2010). An evaluation of the number of lymph nodes examined and survival for node-negative esophageal carcinoma: data from China. Ann Surg Oncol.

[9] Zhu Z, Chen H, Yu W, Fu X, Xiang J, Li H, Zhang Y, Sun M, Wei Q, Zhao W, Zhao K (2014). Number of negative lymph nodes is associated with survival in thoracic esophageal squamous cell carcinoma patients undergoing three-field lymphadenectomy. Ann Surg Oncol.

[10] Hsu PK, Huang CS, Wang BY, Wu YC, Chou TY, Hsu WH (2013). The prognostic value of the number of negative lymph nodes in esophageal cancer patients after transthoracic resection. Ann Thorac Surg.

[11] Wu SG, Li FY, Zhou J, Lin Q, Sun JY, Lin HX, Guan XX, He ZY (2015). Prognostic value of different lymph node staging methods in esophageal squamous cell carcinoma after esophagectomy. Ann Thorac Surg.

[12] Tan Z, Ma G, Yang H, Zhang L, Rong T, Lin P (2014). Can lymph node ratio replace pn categories in the tumor- node-metastasis classification system for esophageal cancer?. J Thorac Oncol.

[13] Wang N, Jia Y, Wang J, Wang X, Bao C, Song Q, Tan B, Cheng Y (2015). Prognostic significance of lymph node ratio in esophageal cancer. Tumour Biol.

[14] Chen YJ, Schultheiss TE, Wong JY, Kernstine KH (2009). Impact of the number of resected and involved lymph nodes on esophageal cancer survival. J Surg Oncol.

[15] Hu Y, Hu C, Zhang H, Ping Y, Chen LQ (2010). How does the number of resected lymph nodes influence TNM staging and prognosis for esophageal carcinoma?. Ann Surg Oncol.

[16] van der Schaaf M, Johar A, Wijnhoven B, Lagergren P, Lagergren J (2015). Extent of lymph node removal during esophageal cancer surgery and survival. J Natl Cancer Inst.

[17] Song YX, Gao P, Wang ZN, Tong LL, Xu YY, Sun Z, Xing CZ, Xu HM (2011). Which is the most suitable classification for colorectal cancer, log odds, the number or the ratio of positive lymph nodes?. PLoS One.

[18] Arslan NC, Sokmen S, Canda AE, Terzi C, Sarioglu S (2014). The prognostic impact of the log odds of positive lymph nodes in colon cancer. Colorectal Dis.

[19] Sun Z, Xu Y, Li de M, Wang ZN, Zhu GL, Huang BJ, Li K, Xu HM (2010). Log odds of positive lymph nodes: a novel prognostic indicator superior to the number-based and the ratio-based N category for gastric cancer patients with R0 resection. Cancer.

[20] Qiu MZ, Qiu HJ, Wang ZQ, Ren C, Wang DS, Zhang DS, Luo HY, Li YH, Xu RH (2012). The tumor-log odds of positive lymph nodes-metastasis staging system, a promising new staging system for gastric cancer after D2 resection in China. PLoS One.

[21] Kim Y, Spolverato G, Amini N, Margonis GA, Gupta R, Ejaz A, Pawlik TM (2015). Surgical Management of Intrahepatic Cholangiocarcinoma: Defining an Optimal Prognostic Lymph Node Stratification Schema. Ann Surg Oncol.

[22] Spolverato G, Ejaz A, Kim Y, Squires MH, Poultsides G, Fields RC, Bloomston M, Weber SM, Votanopoulos K, Acher AW, Jin LX, Hawkins WG, Schmidt C (2015). Prognostic Performance of Different Lymph Node Staging Systems After Curative Intent Resection for Gastric Adenocarcinoma. Ann Surg.

[23] La Torre M, Nigri G, Petrucciani N, Cavallini M, Aurello P, Cosenza G, Balducci G, Ziparo V, Ramacciato G (2014). Prognostic assessment of different lymph node staging methods for pancreatic cancer with R0 resection: pN staging, lymph node ratio, log odds of positive lymph nodes. Pancreatology.

[24] Wang J, Hassett JM, Dayton MT, Kulaylat MN (2008). The prognostic superiority of log odds of positive lymph nodes in stage III colon cancer. J Gastrointest Surg.

[25] Ueno S, Tanabe G, Sako K, Hiwaki T, Hokotate H, Fukukura Y, Baba Y, Imamura Y, Aikou T (2001). Discrimination value of the new western prognostic system (CLIP score) for hepatocellular carcinoma in 662 Japanese patients. Cancer of the Liver Italian Program. Hepatology.

[26] Cho YK, Chung JW, Kim JK, Ahn YS, Kim MY, Park YO, Kim WT, Byun JH (2008). Comparison of 7 staging systems for patients with hepatocellular carcinoma undergoing transarterial chemoembolization. Cancer.

[27] Kee KM, Wang JH, Lee CM, Chen CL, Changchien CS, Hu TH, Cheng YF, Hsu HC, Wang CC, Chen TY, Lin CY, Lu SN (2007). Validation of clinical AJCC/UICC TNM staging system for hepatocellular carcinoma: analysis of 5,613 cases from a medical center in southern Taiwan. Int J Cancer.

[28] Yoon HM1, Ryu KW, Nam BH, Cho SJ, Park SR, Lee JY, Lee JH, Kook MC, Choi IJ, Kim YW (2012). Is the new seventh AJCC/UICC staging system appropriate for patients with gastric cancer?. J Am Coll Surg.

[29] Thomas W, Rice TW Staging of esophageal cancer: TNM and beyond. Esophagus.

